# Transient expression of human interleukin-15 in *Nicotiana benthamiana*

**DOI:** 10.5511/plantbiotechnology.25.0711a

**Published:** 2025-12-25

**Authors:** Yuuka Mukai, Yusuke Taguchi, Kouki Matsuo

**Affiliations:** 1 School of Nutrition and Dietetics, Faculty of Health and Social Work, Kanagawa University of Human Services, 1-10-1 Heiseicho, Yokosuka, Kanagawa 238-8522, Japan; 2 Frontier Business Division, Chiyoda Corporation, 4-6-2 Minatomirai, Nishi-ku, Yokohama, Kanagawa 220-8765, Japan; 3 Biomanufacturing Process Research Center (BPRC), National Institute of Advanced Industrial Science and Technology (AIST), 2-17-2-1 Tsukisamu-Higashi, Toyohira-ku, Sapporo, Hokkaido 062-8517, Japan

**Keywords:** agroinfiltration, interleukin-15, *Nicotiana benthamiana*, plant-made pharmaceuticals, transient expression

## Abstract

A plant-based expression system provides a cost-effective, scalable, and safe alternative to traditional cell culture platforms. In this study, recombinant human interleukin-15 (IL-15) was transiently expressed in *Nicotiana benthamiana* plants using agroinfiltration. IL-15 is a cytokine with significant potential in cell engineering, immunotherapy, and cancer therapy. A codon-optimized IL-15 gene was cloned into a binary vector designed for plant expression and introduced into *Rhizobium radiobacter* (formerly *Agrobacterium tumefaciens*). The *R. radiobacter* for human IL-15 expression was infiltrated into *N. benthamiana* leaves. Following purification, receptor-binding assays confirmed that the plant-derived IL-15 could bind to the IL-15 receptor comparably to its mammalian-produced counterpart. This first report of IL-15 expression in plants highlights the promise of plant-based systems for biopharmaceutical production and lays the groundwork for further development of IL-15 for applications in cell engineering, clinical therapies, and the cultured meat industry.

Plant-based expression systems have emerged as a compelling alternative for recombinant protein production, offering key advantages such as lower production costs, rapid scalability, and reduced risk of contamination from animal-derived pathogens. Employing plants to manufacture recombinant pharmaceutical proteins, including antibodies, cytokines, and vaccine components, presents a promising strategy due to their inherent safety and cost-effectiveness. A notable example is recombinant human glucocerebrosidase, a therapy for Gaucher disease, produced in genetically modified carrot cells and approved by the U.S. Food and Drug Administration (Drake et al. 2017; Traynor 2012). In 2014, patients infected with the Ebola virus were treated with ZMapp, an experimental drug composed of three monoclonal antibodies targeting the Ebola virus, produced in *Nicotiana benthamiana* plants (Lyon et al. 2014). More recently, a plant-based coronavirus disease of 2019 (COVID-19) vaccine containing virus-like particles of the severe acute respiratory syndrome coronavirus 2 (SARS-CoV-2) spike protein, also produced in *N. benthamiana*, received regulatory approval in Canada (Su et al. 2023). Building on these advancements, numerous plant-derived pharmaceutical proteins are currently undergoing clinical trials (Lomonossoff and D’Aoust 2016; Sethi et al. 2021). *N. benthamiana*, a widely used model species in molecular farming, has shown significant promise for the transient expression of high-value biopharmaceuticals through agroinfiltration (Beritza et al. 2024). This method involves introducing recombinant genes into plant tissues via *Rhizobium radiobacter* (formerly *Agrobacterium tumefaciens*), resulting in rapid, transient protein expression in plant cells (Beritza et al. 2024). Numerous pharmaceutical proteins have been successfully expressed in plant systems, supporting the feasibility of plants as a viable platform for producing bioactive proteins (Cao et al. 2022; da Cunha et al. 2014; Das et al. 2022; Venkataraman et al. 2023). For instance, granulocyte-macrophage colony-stimulating factor (GM-CSF) has been expressed in both *N. tabacum* and *N. benthamiana* (Matsuo et al. 2016; Vojta et al. 2015). Interleukin-6 (IL-6) has also been successfully produced in *N. benthamiana*, underscoring the potential of plants for cytokine-based therapeutics (Islam et al. 2019; Nausch et al. 2012). Similarly, various types of interferons have been transiently expressed in plant systems (Heidari-Japelaghi et al. 2020; Xie et al. 2024). In addition, multiple vaccine components have been produced in *N. benthamiana* (Stander et al. 2022). Collectively, these studies demonstrate the versatility and effectiveness of plant-based systems for expressing clinically relevant pharmaceutical proteins.

Interleukin-15 (IL-15) is a cytokine that plays a vital role in regulating immune responses by supporting the development, survival, and activation of immune cells such as natural killer (NK) cells and CD8+ T cells (Duan et al. 2024; Silveira et al. 2022; Vahidi et al. 2024). Owing to its unique properties, IL-15 has gained attention as a promising therapeutic agent for cancer immunotherapy, autoimmune diseases, and vaccine development (Allard-Chamard et al. 2020; Rodríguez-Álvarez et al. 2021; Vahidi et al. 2024). In particular, IL-15 serves as a key adjunct in chimeric antigen receptor (CAR)-T and NK cell therapies (Ghorai and Pearson 2024). It is also recognized as a relevant cytokine in the cultured meat industry (Shaikh et al. 2021). It has been reported that IL-15 stimulates protein synthesis while inhibiting protein degradation in cultured skeletal myotubes (Quinn et al. 1997). Moreover, IL-15 is known to promote myogenesis (O’Leary et al. 2017); therefore, its application in the production of cultured meats may enhance production efficiency. While IL-15 holds great promise, its application requires controlled administration, defined duration, and localized delivery. This underscores the need for a stable, low-cost supply of IL-15. Recombinant human IL-15 has been produced in *Escherichia coli* (Ahmed et al. 2021; Ward et al. 2009), *Pichia pastoris* (Sun et al. 2016), and HEK293 cells (Thaysen-Andersen et al. 2016); however, its expression in plants has yet to be reported.

Here, we successfully expressed biologically active human IL-15 in *N. benthamiana* via a plant-based transient expression system. A codon-optimized IL-15 gene was cloned into a binary vector for plant expression and introduced into *R. radiobacter* for transient expression. This work marks the first successful report of IL-15 expression in plants.

The codon-optimized human IL-15 gene (NCBI accession number P40933), excluding the native signal peptide and propeptide sequences, was fused at the N-terminus with the *N. benthamiana* extensin signal peptide (Jiang et al. 2020) and at the C-terminus with a FLAG tag. The construct was synthesized and subcloned into the pRI201-AN binary vector (TakaraBio, Otsu, Japan; #3264) by Eurofins Genomics (Tokyo, Japan). *R. radiobacter* strain LBA4404, harboring the IL-15 expression construct, was used for agroinfiltration. To enhance protein expression, the pBI121:p19 vector, a pBI121-based construct expressing the artichoke mottled crinkle virus p19 RNA silencing suppressor (Lombardi et al. 2009), was kindly provided by Dr. Benvenuto (ENEA, Italy). Details of *Rhizobium*-mediated green fluorescent protein (GFP) expression were described previously (Matsuo 2022; Matsuo et al. 2016).

*N. benthamiana* plants were hydroponically cultivated in a greenhouse at AIST, Sapporo, Hokkaido, Japan, under controlled conditions at 23–25°C. A hydroponic nutrient solution, Vegetable Life A (OAT AGRIO, Tokyo, Japan), was used to support plant growth. *R. radiobacter* LBA4404 strains harboring the respective expression vectors were pre-cultured overnight at 28°C in yeast extract peptone medium supplemented with kanamycin (50 µg ml^−1^), streptomycin (300 µg ml^−1^), and rifampicin (100 µg ml^−1^) under vigorous shaking. After incubation, bacterial cells were harvested by centrifugation at 5,000×g for 10 min at 23°C and resuspended in infiltration buffer containing 10 mM 2-(N-morpholino)ethanesulfonic acid-KOH (MES-KOH) (pH 5.7), 10 mM MgCl_2_, and 150 µM acetosyringone. The suspension’s optical density (OD_600_) was adjusted to 0.5 and incubated at 23–25°C for a minimum of 2 h before infiltration. For IL-15 expression, the *Rhizobium* suspension was mixed with a separate suspension for p19 expression in a 9 : 1 ratio prior to infiltration. This mixture was infiltrated into the leaves of 4–5-week-old wild-type *N. benthamiana* plants using a 1-ml needleless syringe. GFP, lacking the FLAG tag, was transiently expressed as a negative control (Matsuo 2022). After infiltration, the plants were transferred to a growth chamber maintained at 23°C with a 16-h light/8-h dark photoperiod.

Harvested leaves were ground and homogenized in five volumes (w/v) of binding/washing buffer containing phosphate-buffered saline (PBS, pH 7.2), 0.1% Tween 20, and a protease inhibitor cocktail for plant extracts (Sigma-Aldrich, St. Louis, MO, USA; #P9599). The homogenate was centrifuged at 23,000×g for 10 min at 4°C, and the supernatant was collected for further processing. Anti-DYKDDDDK tag antibody beads (Fujifilm Wako Pure Chemical, Osaka, Japan; #018-22783) were added to the supernatant, followed by centrifugation at 7,000×g for 1 min at 4°C to pellet the beads. The supernatant was discarded, and the beads were washed three times with the same buffer. IL-15 was then eluted using buffer supplemented with FLAG peptide (150 µg ml^−1^, Sigma–Aldrich; #F3290). GFP-expressing leaves were processed in parallel as negative controls.

The purified IL-15 solution was loaded onto a 15% (w/v) SDS-PAGE gel (Atto, Tokyo, Japan). Proteins were transferred onto a polyvinylidene fluoride (PVDF) membrane (Atto) and probed with an IL-15 antibody (E-4) (Santa Cruz Biotechnology, Dallas, TX, USA; #sc-8437) as the primary antibody. Human recombinant IL-15 protein (Thermo Fisher Scientific, Waltham, MA, USA; #200-2-2UG) was used as the standard. A horseradish peroxidase-conjugated sheep anti-mouse IgG antibody (GE Healthcare, Chicago, IL, USA) was used for detection.

For quantification of plant-expressed IL-15, a sandwich enzyme-linked immunosorbent assay (ELISA) was conducted using a commercial Human IL-15 ELISA kit (Proteintech, IL, USA; #KE00102), following the manufacturer’s instructions. Briefly, 100 µl of serially diluted plant-expressed IL-15 samples (prepared using sample diluent) were added to wells precoated with a human IL-15-specific antibody. As references, 100 µl of reconstituted IL-15 standard from the kit and commercial IL-15 (Proteintech; #HZ-1323), also diluted with sample diluent, were added to separate wells. The plate was sealed and incubated for 2 h at 37°C, followed by four washes with wash buffer. Captured IL-15 was detected using 100 µl of biotinylated IL-15-specific antibody (diluted 1 : 100 in detection diluent) incubated for 1 h at 37°C. After washing, 100 µl of streptavidin-HRP conjugate (1 : 100 dilution) was added and incubated for 40 min at 37°C, followed by four additional washes. For signal development, 100 µl of tetramethylbenzidine substrate solution was added and incubated in the dark for 15 min at 37°C. The reaction was terminated by adding 100 µl of stop solution. Absorbance was measured at 450 nm with a correction wavelength of 630 nm using a microplate reader (SH9000Lab; Corona Electric, Hitachinaka, Japan). All measurements were performed in duplicate or triplicate. The concentrations of plant-expressed and commercial IL-15 were calculated using a standard curve equation.

The binding ability of plant-expressed IL-15 to the IL-15 receptor was evaluated using a commercial reporter gene assay kit, IL-15 Bioassay (Promega, Madison, WI, USA; #JA2011), following the manufacturer’s instructions. The assay employs genetically engineered IL-15 bioassay cells, which produce a luminescent signal upon receptor-mediated pathway activation when IL-15 binds to its receptor. Luminescence is triggered by the addition of the Bio-Glo Reagent and quantified using a luminometer. Briefly, IL-15 bioassay cells were retrieved from storage at −150°C and thawed gently with light agitation. A total of 0.8 ml of cells was transferred into a 15 ml conical tube containing 7.2 ml of prewarmed assay buffer and mixed gently by pipetting. Using an electronic multi-dispenser pipette, 50 µl of the cell suspension was dispensed into each well of white, flat-bottom 96-well assay plates (Thermo Fisher Scientific; #136101). The plates were then covered and incubated at 37°C in a 5% CO_2_ humidified incubator while samples and dilutions were prepared. Serial dilutions of both recombinant plant-expressed IL-15 and commercial IL-15 were prepared post-plating. Starting from a 100 ng ml^−1^ stock, each IL-15 was first diluted 10-fold and then subjected to 1.8-fold serial dilutions. To prepare these, 120 µl of assay buffer was dispensed into wells of a sterile clear 96-well plate. Then, 150 µl of the 10 ng ml^−1^ IL-15 solution was added to the first well and mixed by gentle pipetting. Subsequently, 150 µl was transferred from one well to the next across 10 wells to generate a dilution series, resulting in IL-15 concentrations ranging from 50 to 10,000 pg ml^−1^. Each dilution (120 µl) was sufficient for triplicate analysis. As a negative control, the purified product from GFP-expressing plants was similarly subjected to 1.8-fold serial dilution in parallel with recombinant IL-15.

For the stimulation assay, 25 µl of each dilution or assay buffer (as a no-drug control) was added to the 50 µl of plated cells in duplicate or triplicate. Then, the assay plates were recovered with a lid and incubated in a 37°C, 5% CO_2_ humidified incubator for 6 h. After incubation, the assay plates were removed from the incubator and equilibrated to ambient temperature for 10 min. 75 µl of prepared Bio-Glo Reagent was added to all test wells and blank wells to measure the background signal. After incubation at ambient temperature, luminescence was measured using the SH-9000Lab luminescence plate reader (Corona Electric). As the luminescence of the no-drug control was at least 100× higher than the plate background, background subtraction was deemed unnecessary. Data were plotted as luminescence versus log_10_ [sample concentration], and EC_50_ values for the IL-15 response were calculated from fitted curves and normalized to the IL-15 concentration.

The codon-optimized IL-15 gene, containing a C-terminal FLAG tag and an N-terminal extensin signal peptide from *N. benthamiana*, was subcloned into the binary vector pRI201-AN (Figure 1). In this construct, the propeptide region was omitted. For transient expression in *N. benthamiana*, the vector was introduced into *R. radiobacter* strain LBA4404. Cultures were grown in yeast extract peptone (YEP) medium with antibiotics, harvested by centrifugation, and resuspended in MES buffer. Agroinfiltration was performed using a needleless syringe to introduce the bacterial suspension into the leaves. Infiltrated tissues were harvested five days post-infiltration. IL-15 protein was purified by affinity-based purification using anti-FLAG antibody beads. Western blotting confirmed the presence of plant-expressed IL-15 (estimated molecular weight: 16.4 kDa), with specific bands indicating successful expression (Figure 2). Interestingly, Western blotting with an anti-IL-15 antibody revealed two bands for both plant- and *E. coli*-expressed IL-15. The *E. coli-*expressed IL-15 used in this experiment lacked a signal peptide, so the appearance of two bands is unlikely due to signal peptide cleavage. Moreover, since proteins expressed in *E. coli* typically lack glycosylation, this also is an unlikely cause for the double bands. IL-15 has been reported to form intramolecular disulfide bonds (Lowe et al. 2011), suggesting that incomplete reduction during sample preparation may have led to the simultaneous detection of both reduced and non-reduced forms of IL-15. In two independent expression experiments, IL-15 yields were 0.58 µg g^−1^ fresh weight (IL-15_ex1) and 4.15 µg g^−1^ fresh weight (IL-15_ex2), as quantified by ELISA. The approximately 7-fold difference in IL-15 expression between the two experiments may be attributed to seasonal variations, as the plants were grown in the greenhouse under different environmental conditions. It has been reported that recombinant protein production can fluctuate depending on the plant’s growth environment (Fujiuchi et al. 2016; Knödler et al. 2019).

**Figure 1:**
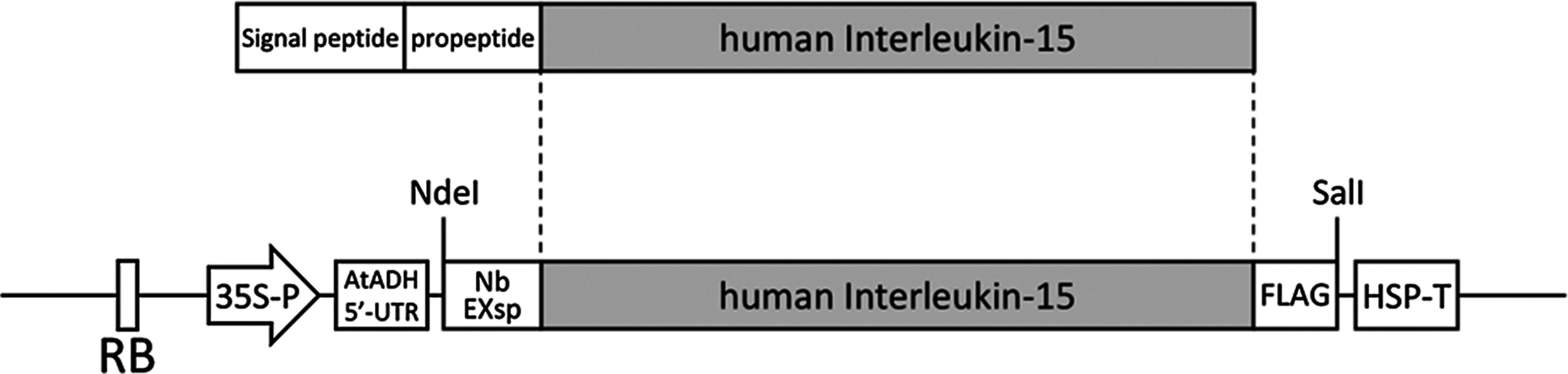
Figure 1. Expression cassette of human IL-15. The human IL-15 gene, fused at the N-terminus with the signal peptide of extensin from *Nicotiana benthamiana* and at the C-terminus with a FLAG tag, was subcloned into the multiple cloning site of the pRI201-AN binary vector. RB, right border sequence; 35S-P, cauliflower mosaic virus 35S promoter; AtADH 5′-UTR, 5′-untranslated region of alcohol dehydrogenase from *Arabidopsis thaliana*; NbEXsp, signal peptide of extensin from *N. benthamiana*; FLAG, FLAG tag sequence; HSP-T, terminator sequence of heat shock protein from *A. thaliana*.

**Figure 2:**
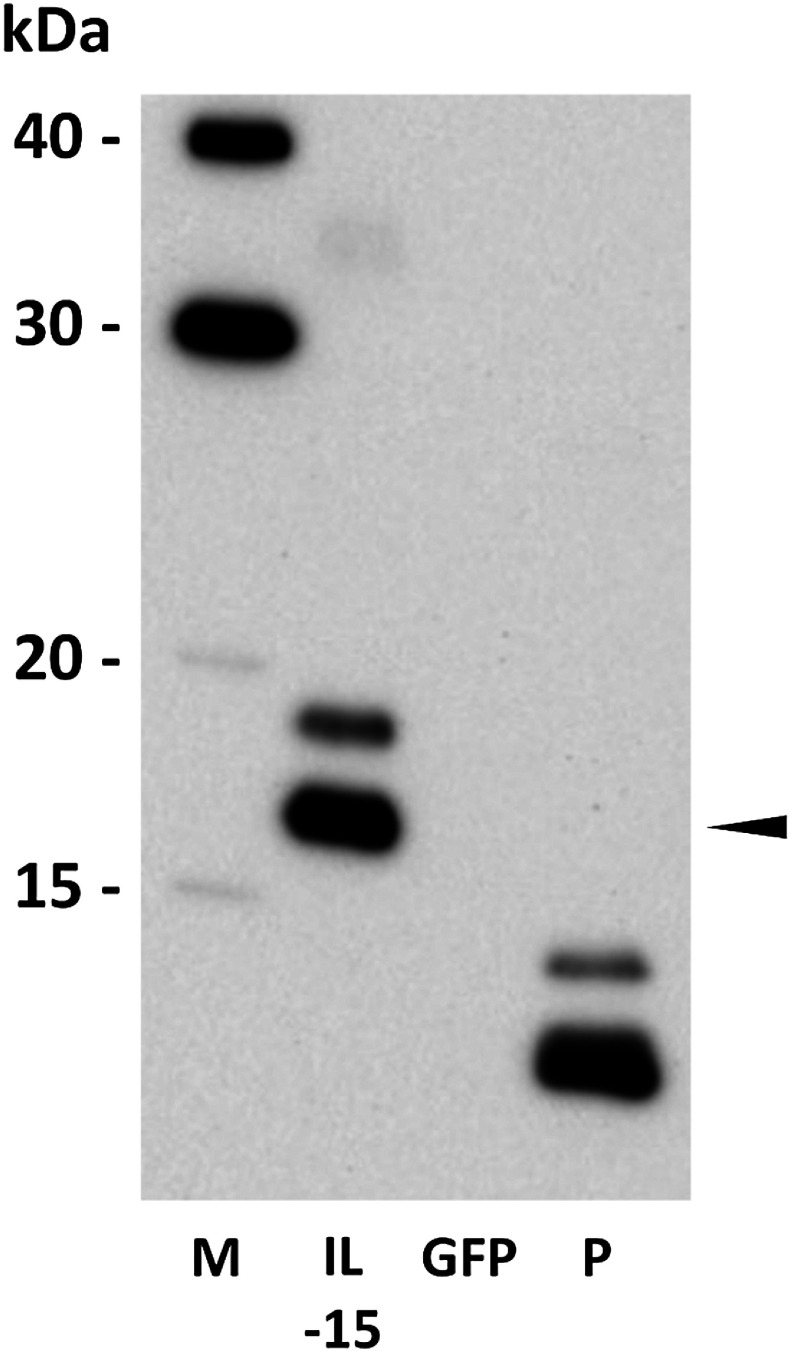
Figure 2. Western blot analysis of plant-expressed IL-15. IL-15 expressed in the leaves of *Nicotiana benthamiana* was affinity-purified using agarose beads conjugated with an anti-FLAG antibody. GFP-expressing plants were processed in the same manner as controls. Recombinant IL-15 was detected using an anti-IL-15 antibody as the primary antibody. The arrow indicates plant-expressed IL-15. M, protein size marker; IL-15, purified product from human IL-15-expressing plants; GFP, purified product from GFP-expressing plants; P, *Escherichia coli*-expressed human IL-15 (estimated molecular weight is 12.9 kDa).

To evaluate the receptor-binding ability of plant-expressed IL-15, IL-15 bioassay cells expressing the specific IL-15 receptor on their surface were used. Binding assays were conducted using two independently produced plant-derived IL-15 samples (ex1 and ex2), commercial IL-15, and purified products from GFP-expressing leaves as a negative control. Plate background relative light units (RLUs) were negligible. As shown in Figure 3, RLUs generated by both plant-derived IL-15 ex1, ex2 and the commercial IL-15 were detected, whereas RLUs from the GFP control remained at the baseline concentration across all concentrations. Little difference was found between IL-15_ex1 (EC_50_=538 pg ml^−1^) and IL-15_ex2 (EC_50_=540 pg ml^−1^). The commercial IL-15 demonstrated a slight improvement in efficacy (EC_50_=454 pg ml^−1^). These findings suggest that the plant-expressed IL-15 possesses receptor-binding ability comparable to that of the human-derived commercial IL-15.

**Figure 3:**
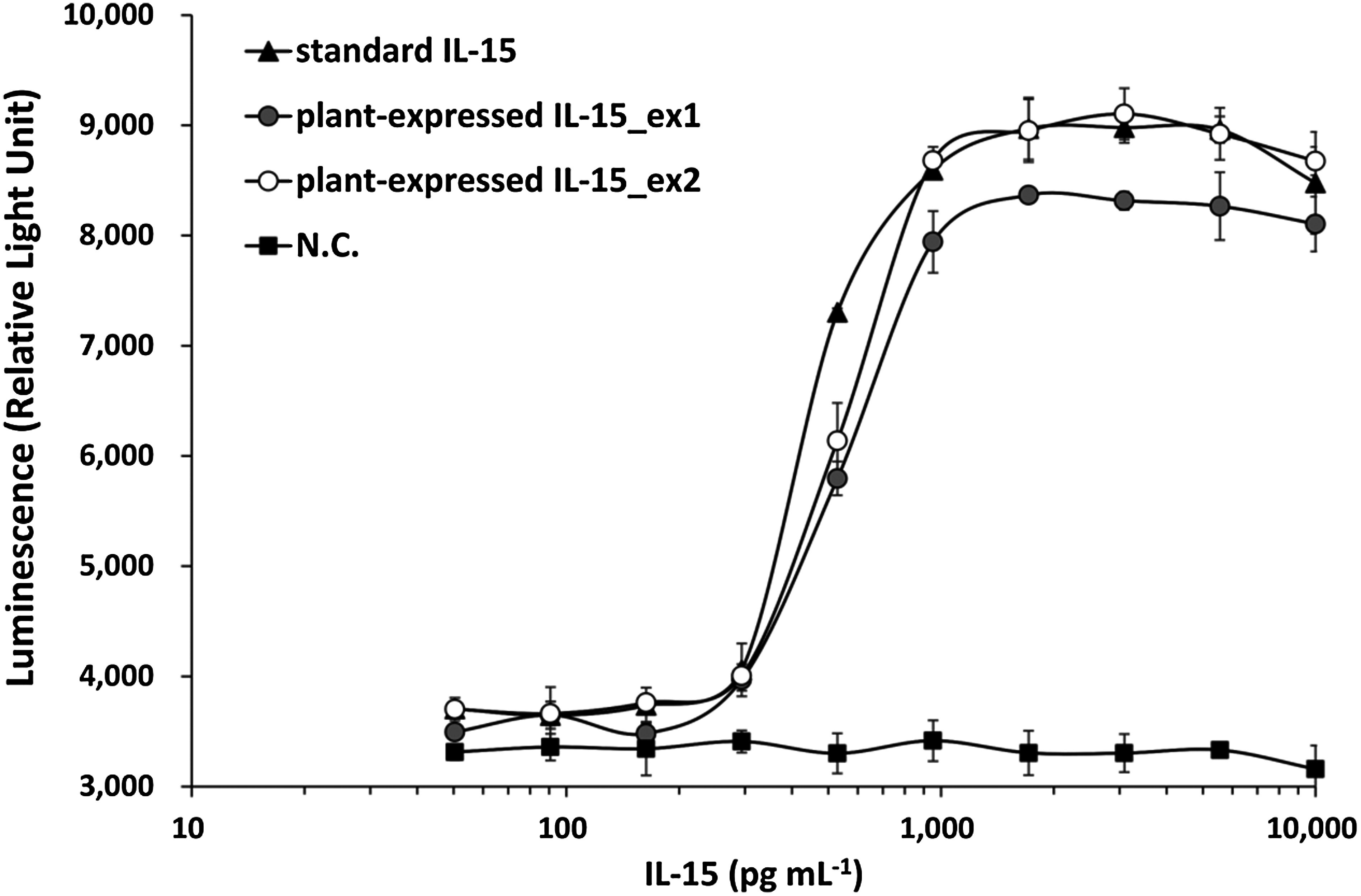
Figure 3. Receptor-binding assay of plant-expressed IL-15. The binding ability of plant-expressed IL-15 to its receptor was evaluated using the IL-15 Bioassay (Promega). Upon receptor binding, IL-15 activates a signaling pathway that induces luminescence, which was detected and quantified using a luminometer. Data are presented as mean±SD [two technical replicates for standard IL-15, plant-expressed IL-15_ex1, and negative control (N.C.); three technical replicates for plant-expressed IL-15_ex2]. N.C., negative control (purified product from GFP-expressing leaves).

One of the major barriers to the application of cell therapies and the commercialization of cultured meat is the high cost of media, particularly the need to include growth factors and cytokines (Enriquez-Ochoa et al. 2020; Xie and Murphy 2019). To lower media costs and enhance safety, serum-free media not only circumvent ethical concerns but also improve the consistency and reliability of the production process. The development of serum-free media is also anticipated to significantly boost the sustainability of the cultured meat industry (Lee et al. 2022). However, even serum-free media require the supplementation of certain growth factors (Ritacco et al. 2018), leaving the challenge of cost reduction unresolved; nonetheless, these factors remain crucial for establishing a sustainable and economically viable cell culture system. Plant expression systems have also been employed for the production of various cytokines, further demonstrating their capacity to produce functionally active therapeutic proteins. (Cao et al. 2022; da Cunha et al. 2014; Das et al. 2022).

Recombinant IL-15 capable of binding the IL-15 receptor was successfully expressed in *N. benthamiana* via agroinfiltration in the present work. The binding ability of plant-expressed IL-15 to the IL-15 receptor was comparable to that of commercially available IL-15, highlighting the potential of plants for recombinant protein production. Plant-derived IL-15 may help reduce the cost of producing cells for therapies such as CAR-T therapy. As IL-15 also plays a critical role in myogenesis (Shaikh et al. 2021), it is anticipated to lower the cost of cultured meat production. Additionally, since IL-15 has been reported to be effective in cancer treatment (Isvoranu et al. 2021), plant-produced IL-15 may contribute to more affordable cancer therapies. For future medical applications, a more detailed evaluation of the bioactivity of plant-expressed IL-15 will be necessary. It has been reported that a heterodimer composed of IL-15 and its receptor exhibits greater activity in vivo than the IL-15 monomer (Chertova et al. 2013). Recently, NIZ985, a recombinant heterodimer of physiologically active IL-15 and IL-15 receptor α, has entered Phase I clinical trials (Conlon et al. 2021). Therefore, it would also be interesting to explore the expression of IL-15 receptors in plants.

In this study, the yield of plant-produced IL-15 was 0.58 and 4.15 µg g^−1^-fresh weight, comparable to the expression levels of other cytokines in plants (da Cunha et al. 2014). In contrast, *E. coli* and yeast have been reported to express 120 and 75 mg l^−1^ of recombinant IL-15, respectively (Ahmed et al. 2021; Sun et al. 2016). Therefore, the expression level of IL-15 in this study remains lower and requires further optimization to match the yields obtained in *E. coli* and yeast. To enhance the expression of recombinant proteins in plants, several strategies—such as enhancement of subcellular localization of recombinant proteins, codon optimization, promoter and terminator engineering, and reduction of recombinant protein degradation-have been reported (Beritza et al. 2024; Feng et al. 2022). To enhance mRNA stability in plant cells, we employed codon optimization along with co-expression of an RNA silencing suppressor protein. Applying additional methods described above may further increase IL-15 expression.

## Abbreviations

ELISA: enzyme-linked immunosorbent assay
GFP: green fluorescent protein
IL-15: interleukin-15
NK cells: natural killer cells
